# Kidstime workshops: the evaluation of a multi-family intervention for children of parents with mental illness

**DOI:** 10.1007/s00787-025-02853-z

**Published:** 2025-08-27

**Authors:** Esther Strittmatter, Niklas Helsper, Jens Joas, Alan Cooklin, Eva Möhler, Klaus Henner Spierling

**Affiliations:** 1 Department of Child and Adolescent Psychiatry, Faculty of Medicine, Saarland University, 66421 Homburg, Germany; 2 Integration Assistance and Healthcare Department, Institute for Child and Youth Welfare (IKJ), Essen, Germany; 3 Founder of the Kidstime/OurTime Foundation, London, UK; 4 Social Paediatric Center, Agaplesion Diakonie Hospital, Rotenburg, Germany

**Keywords:** Kidstime_1_, Children of parents with mental illness_2_, Prevention_3_, Therapy_4_, Resilience_5_

## Abstract

**Background:**

Kidstime workshops were conceptualized as a low-threshold intervention for children of parents with mental illness (COPMI).

**Methods:**

Kidstime workshops were set up at eleven locations throughout Germany. The multi-center eligibility study employed a pre-post-design. In addition to selected capability items, clinical scales were analyzed.

**Results:**

The preliminary results demonstrated significant improvements in the children’s distress and impairment as well as improvements in parental psychopathology and psychosocial functioning. There were also improvements in capability-based measures of psychological integrity and resilience, participation, life motivation and satisfaction in both children and parents. Our analysis suggests that Kidstime workshops can be more effective at improving capabilities, with better resource-benefit ratio compared with nationwide data of general child support interventions.

**Conclusion:**

The preliminary results suggest that Kidstime workshops show therapeutic potential by improving wellbeing and participation for both COPMI and their parents. The Kidstime workshops address an important gap in support for COPMI by implementing a multi-family-based approach in the families’ social environment and providing cross-system delivery with low costs and few hours of investment.

**Supplementary Information:**

The online version contains supplementary material available at 10.1007/s00787-025-02853-z.

**Word count**: 5986.

## 1. Introduction

The 12-month prevalence of disorders of mental health in adults in Germany is 27.8% [1], which means that 17.8 million people are affected. Approximately 3.8 million children and young people in Germany live with a parent with a mental illness. These children are at high risk for developmental threats, adverse childhood experiences and childhood trauma [2]. Without appropriate support it is estimated, that 41–77% of these young people develop mental disorders during their lives [3, 4]. Although results vary between studies, a meta-analysis indicated that offspring of parents with severe mental illness had an increased risk of a range of psychiatric disorders and one third of them may develop a severe mental illness by early adulthood [5]. In addition to an increased incidence of mental and somatic diseases, more severe courses of illness and increased mortality have been observed [6–8]. Intergenerational transmission of mental illness constitutes a personal and socioeconomic risk factor [9]. Therefore, prevention, early detection and tailored therapeutic interventions are of personal, social and economic importance to interrupt transgenerational transmission of mental disorders.

The aim of the present study was to evaluate the effects of Kidstime workshops on psychopathology and psychosocial functioning from a multi-perspective view. In addition, the relative efficiency compared to other interventions for children of parents with mental illness (COPMI) should be investigated.

Kidstime workshops were designed to address COPMI and their particular needs [10]. Primary goals were to

1. provide understandable explanations for the children about the parents’ illness,
2. reduce children’s fears, insecurities and confusion,
3. help parents talk to their children about their illness and its effects,
4. help parents rediscover and strengthen their trust, confidence, skills and dignity,
5. reduce stigmatization, isolation and loneliness,
6. create an environment for growth and hope,
7. promote positive parent‒child interaction,
8. foster creativity and playfulness using play, drama and filming.
9. encourage children to engage in age-appropriate, joyful activities [11–13].

The workshop consisted of multi-family groups of up to ten families who met once a month for three hours with a multidisciplinary team of at least four practitioners. To reduce barriers to treatment, workshops were held in local community settings. The workshops included:

- a short social meeting with beverage and snacks upon arrival,
- a psychoeducational workshop with the whole group on specific aspects of mental illness and support and treatment options,
- separate groups for parents and children and young people of about one hour. In the children’s group this usually started with fun games, followed by drama activities, like filming or drawing of young peoples’ stories – if possible linked to the preceding psychoeducational input. In the parents’ group the focus was on joint discussions of ways to manage parental responsibilities, whilst coping with a mental illness,
- a joint meeting with all participants- parents and children- with Pizza supplied, reporting back from the parents’ group, as well as viewing of the children’s work, followed by appreciating and reflecting on the children’s work.

The Kidstime workshops were conceptualized as a “social event” for prevention, not therapy. However, could they also have therapeutic effects?

The present research aimed to find preliminary answers to this question. While prior research on Kidstime workshops had focused mainly on the effects on participating children [10], the present study also examined possible changes in the well-being of parents. Furthermore, Kidstime workshops were compared to other prevention programs, i.e. the state program KIPS prevention North-Rhine Westphalia (KIPS-NRW) and to educational support from child and youth welfare organizations in terms of their input, effects and costs.

## 2. Materials and methods

### 2.1. Design, data collection and measures

With the funding of the BMG, the Kidstime approach was set up at eleven locations throughout Germany. For the selection of the Kidstime locations, different social contexts (large city, small town, rural community) and different federal states were considered. The main strand of the investigation was a quantitative impact evaluation, which was designed as a “pre-post design”. Data were collected from parents and practitioners involved in the project before (t0) and 6 months after the beginning of the intervention (t1).

In addition to sociodemographic characteristics, the following assessment tools were used for pre- and post-evaluation:

1. The “capability approach” [14] focuses on the capabilities (and competencies) of parents and children for a successful life. The impact-oriented capability scale comprised a total of 16 capability dimensions. Although rarely employed in the field of medicine, a short set of the *Capability Scale* was used, as it allowed a quasi-experimental study design with nationwide comparative data of educational assistance provided by child and youth welfare. It could also be compared with other interventions for COPMI in Germany which were evaluated by the Institute for Child and Youth Welfare (IKJ). This innovative method is currently seen as being a “state of the art” in social-pedagogic discourses [15], as it allows multidimensional change measurement from a multi-perspective view.

For the present study, the following four capability dimensions (abbreviated as “Cap”) were key target areas:

- Cap1: Life motivation and satisfaction (e.g., drive, ability to structure oneself).
- Cap 2: Physical integrity (e.g., physical health, health-related behaviour).
- Cap 3: Psychological integrity and resilience (e.g., psychological stability, emotional experience, tolerance of stress).
- Cap 10: Participation in community and society (e.g., participation in school, community or working life).

Other capability dimensions (such as housing, mobility, practical reasoning, values, leisure and recreation) were considered less important for the present study. They were also omitted for methodological reasons (risk of overfitting and decreasing generalizability, multi-collinearity and interactions).

2) The extended version of the *Strengths and Difficulties Questionnaire (SDQ)* in the three-subscale division was used. Furthermore, the impact of the problem and resulting impairment (chronicity, distress, social impairment, and burden to others) were assessed [16]. The scale was completed by the parents of the participating children (age 4–17). It shows acceptable internal consistency.
3) The *“Brief Symptom Checklist” (BSCL)* is the short version of the SCL-90 and is based on the American ‘Brief Symptom Inventory’. It consists of 53 items covering nine symptom dimensions and three global indices of distress. Among the three global indices, the Global Severity Index (GSI) is the most sensitive indicator of respondents’ distress levels and combines information on the number of symptoms and the intensity of distress. The reliability of the global score is said to be very good [17].
4) The *“Global Assessment of Functioning” (GAF)* is a numeric scale of Axis V of the Diagnostic and Statistical Manual of Mental Disorders, Fourth Edition Text Revision. It briefly illustrates the severity of illness in a generic rather than a diagnosis-specific way. Scores range from 100 (extremely high functioning) to 1 (severely impaired). Therefore, it rates psychological, social, and occupational functioning, covering the range from positive mental health to severe psychopathology [18]. The GAF has acceptable internal consistency (with a Cronbach’s alpha of 0.74).
5) The *“Clinical Global Impressions” (CGI)* scale is well established, easy to use and applicable to all psychiatric disorders [19]. The CGI comprises two companion one-item measures evaluating (a) the severity of psychopathology (*CGI-S*everity) and (b) changes from the initiation of treatment (*CGI-I*mprovement). Compared with the baseline evaluation, the CGI-I assesses improvement on a seven-point scale.

As part of the project, two focus group discussions were conducted with all team leaders of the Kidstime workshop sites at the beginning and at the end of the project. These discussions aimed to provide qualitative insights into the structural, institutional, and case-related success factors, as well as into the challenges in implementing the Kidstime workshop model in Germany. The discussions were documented via detailed protocols and supplementary audio recordings to ensure accuracy and traceability in the data analysis.

### 2.2. Recruitment and participants

**Fig. 1:**
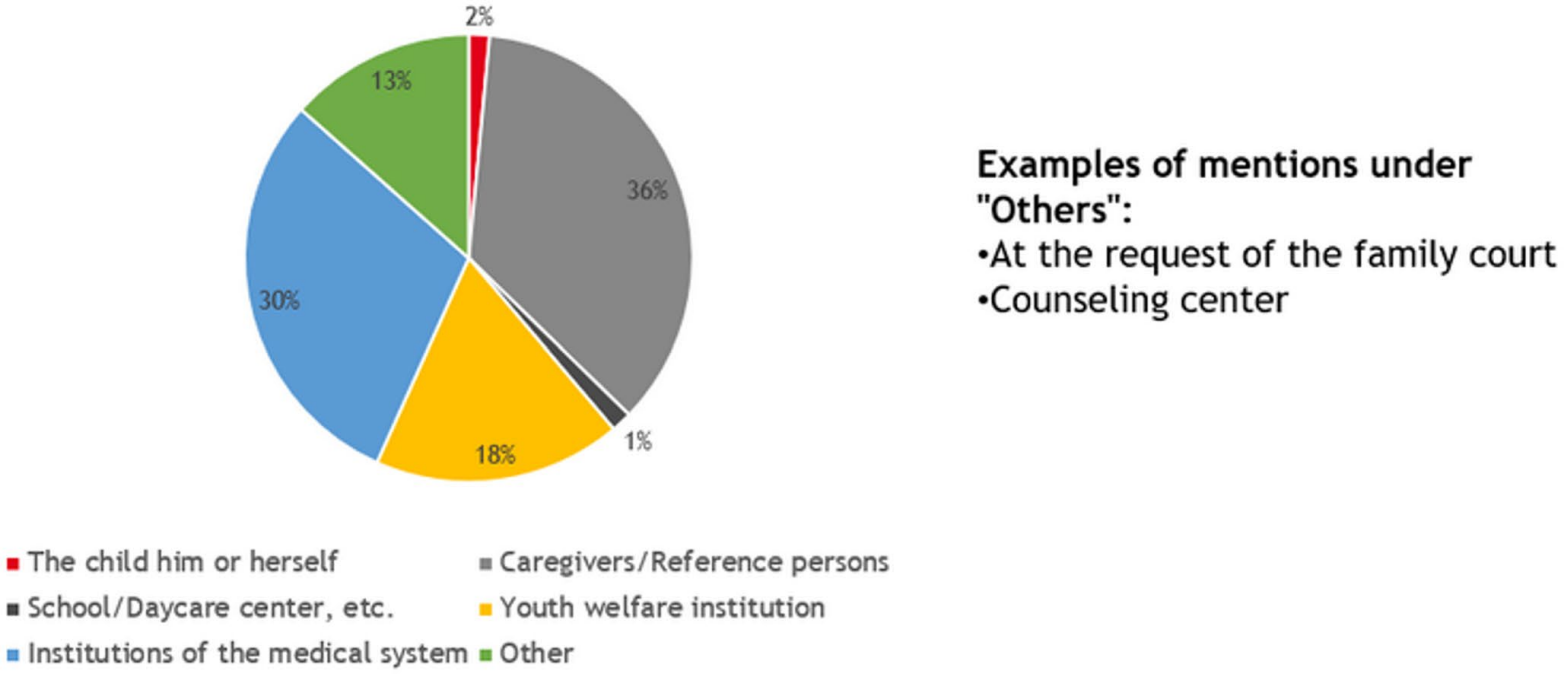
Access path to Kidstime

All families with at least one parent with mental illness were eligible to participate in the study. The only exclusion criteria were acute suicidality, psychosis and intoxication. All children of the respective parents were welcome. Other attachment figures were invited to join the Kidstime workshops as well. The families were recruited through announcements in the media and through existing professional networks. Figure 1 shows the access paths to the Kidstime workshops.

Overall, 85 parents and 103 children (60 boys, 43 girls) participated in the Kidstime workshops. Eleven adults had no evidence of mental illness and thus could not take part in the study. All others were given the opportunity to take part in the evaluation of the Kidstime workshops. 59 parents (80%) with 60 children (33 boys, 27 girls) agreed to participate in the study at t0. The parental mean age was 40.4 years (range: 22–58 years). The mean age of the children was 7.4 years (range: 0–16 years) [see Supplement 1 Table 1].

Eighteen children had no siblings, 38 had one sibling, and three had two siblings. The children’s assessment was carried out by mothers in 76% of the cases (*n* = 45) and by fathers in 24% of cases (*n* = 14). The main diagnoses of the parents were depressive disorder, personality disorder, anxiety disorder, posttraumatic stress disorder and substance misuse. Comorbidities were present in 89% of the patients. A total of 36.6% of the children had a psychiatric diagnosis. The main diagnoses of the children were attention deficit hyperactivity disorder, conduct disorder, emotional disorders with onset specific to childhood and anxiety disorders. An overview of all the diagnoses can be found in Supplement 1 Table 2.

### 2.3. Nationwide comparative data

The data set used for the evaluation of KIPS NRW included data of 75 children and young people who participated in the program, with corresponding inputs from 95 professionals. Complete pre- and post-evaluation data (t0 and t1 after 6–12 months, on average 8.7 months) were available for 31 children. The average age of the children participating in the KIPS NRW program was 9.6 years (SD = 2.9, range: 5–14 years).

Data from educational inputs from child and youth welfare organizations were extracted from the “EVAS – Evaluation erzieherischer Hilfen^1^” system, which focused on interventions provided under § 31 (nonresidential educational support) and § 34 (residential educational support) of the German Social Code (SGBVIII). Our analysis was limited to cases with complete datasets, including both professional and child perspectives, collected at two time points (t0 and t1).

A special feature of the Kidstime workshops was the involvement of the entire family. Since parents were not actively involved in the nationwide comparative data of the IKJ (KIPS NRW, EVAS) and therefore no evaluation from the parents’ perspective took place in these interventions, the parents’ perspective was not included in the calculation of the results in Table 3 for reasons of comparability.

Participants of the § 31 Interventions: Total cases in dataset: 268; cases with complete data (professional & child): 112; age of children: mean = 10.4 years (SD = 3.2; range: 5–17 years); gender distribution: 52% male, 48% female.

§ 34 Interventions: Total cases in dataset: 4,867; cases with complete data (professional & child): 238; age of children: mean = 12.1 years (SD = 2.8; range: 6–18 years); gender distribution: 58% male, 42% female.

### 2.4. Statistical analysis

The evaluation was carried out independently by IKJ. Data was analyzed with IBM SPSS Statistics, version 28. The repeated-measures tool MANOVA was used in our evaluation to identify potential differences. With respect to the MANOVA requirements, 7 extreme values were excluded from the parental data. There was no linear relationship in the SDQ. No transformation was carried out; instead, a decrease in statistical power was accepted, and the calculation continued, since the deviation was not that strong. Multivariate normality of residues was not given in any of the MANOVAs performed, but MANOVA is relatively robust against this violation of assumption when the sample size is reasonably large [20]. Multicollinearity was present in the BSCL: Pearson r-correlation 0.95 PST-T with GSI-T, PSDI-T with GSI-T 0.91. Therefore, only GSI-T was included in the calculations. A significance level of 0.05 was used for all the statistical tests, and all the results were rounded to two decimal places. For GAF, one ANOVA with repeated measures was performed. All the requirements were met. For the change in CGI, a nonparametric test for binomial distribution was calculated with two-sided significance.

For comparison of Kidstime workshops to other kinds of support for COPMI [Kips NRW, EVAS], mean changes, standard deviations, mean hours of investment and mean costs were calculated.

## 3. Results

Table 1 shows the changes of the capability scales in children and parents and changes of GAF in parents according to the professionals’ point of view. Our MANOVAs revealed statistically significant differences in professionals’ views with respect to both children’s overall Cap and caregivers’ overall Cap. Considering children’s capability, scales 1 and 3 had significant effects. Cap 2 was marginally significant. There was also a significant main effect in the ANOVA with repeated measures for the GAF. According to Cohen [21], Cap 3 (children), Cap 1 (parents) and GAF (parents) had large effects, whereas Cap 1 (children) and Cap 2 (parents) had medium effects. With respect to changes in the CGI scores of the parents, there was a significant difference between improvements (*n* = 30) and unchanged or worse conditions (*n* = 4; *p* <.001).

**Table 1:** Results of repeated-measure (M)ANOVA(s): changes of the capability scales in children and parents according to the **professionals point of view**, changes of GAF in parents *p <.05, **p <.01, ’*p* =.051

	*N*		T0		T1									
	T0 & T1 part taken and completed	Excluded outliers	M	SD	M	SD	F	p	Wilk’s *Λ*	Partial *η²*	Post-hoc ANOVA F	p	Partial *η²*	Effect size f
Children														
Cap 1	32	0	3.34	0.90	3.81	0.74	8.45	< 0.01**	0.45	0.55	4.36	< 0.05*	0.12	0.36
Cap 2			3.84	0.92	3.91	1.20					0.06.	0.81	-	-
Cap 3			2.69	1.15	3.91	1.20					29.79	< 0.01**	0.49	0.83
Cap 10			3.50	1.05	3.72	0.96					0.75	0.39	-	-
Caregiver														
Cap 1	31	0	2.81	1.17	3.39	0.62	2.87	0.04*	0.70	0.30	7.55	0.01*	0.20	0.47
Cap 2			3.06	1.18	3.58	0.85					4.15	0.05‘	0.12	0.36
Cap 3			3.10	,83	3.13	0.62					0.04	0.84	-	**-**
Cap 10			3.45	0.93	3.35	1.05					0.33	0.57	-	**-**
GAF	42	0	58.95	10.75	63.24	10.94	8.80	< 0.01**	0.82	0.18	8.80	< 0.01**	0.18	0.45

Table 2 shows the results from the parents’ point of view. (M) ANOVAs detected significant differences in overall Cap as well as in BSCL and SDQ. Furthermore, there were significant effects for improvement in Cap 1 and Cap 2 (parents), Cap 3 (parents and children), Cap 10 (parents and children), BSCL-GSI (parents) and SDQ-Impairment (children). According to Cohen (1988), parents Cap 2 and Cap 10 and children Cap 3 had medium effects. All further effect sizes were large.

**Table 2:** Results of repeated-measure (M)ANOVA(s): changes of the capability scales in children and parents according to the **parents’ point of view** *p <.05, **p <.01

	*N*			T0		T1									
	T0 & T1 part taken	Excluded outliers	T0 & T1 completed	M	SD	M	SD	F	p	Wilk’s *Λ*	Partial *η²*	Post-hoc ANOVA F	p	Partial *η²*	Effect size f
Parental self-assessment															
Cap 1	59	0	50	2.62	1.19	3.24	0.89	2.29	0.04*	0.70	0.30	15.75	< 0.01**	0.24	0.52
Cap 2				2.04	1.29	2.46	1.27					4.07	< 0.05*	0.08	0.29
Cap 3				1.96	1.26	2.62	1.16					10.14	< 0.01**	0.17	0.43
Cap 10				3.06	1.13	3.46	1.25					5.16	0.03*	0.10	0.32
BSCL-GSI T		1	46	64.57	7.20	60.67	8.38	12.28	< 0.01**	0.79	0.21	12.28	< 0.01**	0.21	0.49
Parental assessment of their children															
SDQ total problem score	59	2	33	13.64	6.35	13.21	6.14	4.03	0.03*	0.79	0.21	0.16	0.69	-	-
SDQ impairment				1.58	1.56	0.79	0.89					8.24	< 0.01**	0.21	0.49
Cap 1		1	55	3.40	1.15	3.65	0.97	3.19	0.02*	0.80	0.20	2.90	0.10	-	-
Cap 2				3.36	1.08	3.60	1.10					2.03	0.16	-	-
Cap 3				2.29	1.32	2.75	1.08					6.04	0.02*	0.10	0.32
Cap 10				3.42	1.21	3.89	1.05					9.80	< 0.01**	0.15	0.40

Table 3 compares the two preventive interventions with nonresidential and residential forms of educational support. On the one hand, the number of cases (n) with the mean changes (ΔM) and standard deviation (SD) of the changes in the capacity scales (Cap 1, 2, 3, and 10) were shown. On the other hand, the intervention hours and costs per case in the different interventions were calculated.

For comparability of Kidstime workshops with the representative comparative data of the IKJ, the degree of expression of the basic capabilities (using the six-point response scale) was transformed to a value range from 0 (not at all present) to 100 (fully present) and presented as an overall value across the individual perspectives. Positive values indicated an improvement in capabilities, while negative values indicated a reduction in capabilities. The maximum possible value range was from + 100 to −100.

The following statistical guidelines applied to the interpretation of the absolute level and the difference between values in Table 3: significant large effects were assumed from a mean change of approximately 13.5 points. Significant medium effects were present from a mean change of approximately 8.5 points. Significant small effects were present from a mean change of approximately 3.5 points. Below a value of 3.5 points, there was no statistically significant difference [22].

**Table 3:** Comparison of kidstime with Kips NRW and educational support

Intervention		Kidstime	Kips NRW	Nonresidential educational assistance (§ 31 SGB VIII)	Residential educational assistance (§ 34 SGB VIII)
Change measurement of					
• Cap 1	N	54	29	268	4867
	ΔM	5.31	7.93	2.7	−2.7
	SD	17.98	19.53	21.73	19.35
• Cap 2	N	53	31	268	4862
	ΔM	3.87	7.10	−0.4	−2.7
	SD	15.46	20.36	26.64	18.18
• Cap 3	N	54	28	268	4869
	ΔM	14.23	12.14	4.3	−0.4
	SD	21.67	26.30	16.13	18.44
• Cap 10	N	50	31	267	4866
	ΔM	8.1	6.45	3.2	−1.6
	SD	22.6	16.44	27.45	23.73
Intervention hours per case per professional per year		3	10.5	180	unknown
Costs per case per year (€)*		2000 per family with 1–3 children	approximately 3000 per child	8.970,85 per child	44.165,81 per child

Distribution analysis of the data sets of MANOVA and Table 3 are shown in Supplement 2. Information from the research network of the German Youth Institute and the Technical University of Dortmund was used to calculate the costs of educational assistance. A total sum of € 5.343.957.000 was provided for 121.005 cases in 2022 in relation to help according to § 34 SGBVIII, which resulted in costs of €44.165,81 per case per year. For non-residental educational assistance, a total sum of € 1.247.373.000 was provided for 139.113 cases in 2022, which resulted in costs of €8.970,85 per case per year. The costs of Kidstime workshops were approximately 20 000€ per year. This included staff costs, room rent, materials for the workshops as well as the costs of pizzas and beverages provided for the families. By contrast, KIPS NRW sessions were held weekly, excluding school holidays and cancellations, resulting in approximately 35 sessions per year in the region. Each session lasted 1.5 h and involved an average of 5 children. The sessions were facilitated by two professionals. The cost per session was approximately €1,500. For non-residential educational assistance approximately 180 h of specialist services per year were assumed.

## 4. Discussion

There is ample empirical evidence of the negative effects of parental mental illness on children’s individual and interpersonal functioning [4]. According to Döhnert and Wiegand-Grefe (2020) “the preventive and therapeutic interventions now available show inadequate efficacy to effectively improve the situation” for this high risk population [23]. Research mainly focused on individual psychiatric symptoms and/or diagnoses as outcome variables. Therefore, future interventions should also address the intrafamilial and contextual level [24]. Through the multi-family approach and the integration into the local community setting Kidstime workshops offer the opportunity to include all these levels. Furthermore, our capability focused evaluation tried to broaden the scope towards a broader understanding of wellbeing and participation in a more progressive, human-rights-based model of care [14]. The present study was designed as a feasibility study not as a randomized controlled trial. A major limitation is that blinded raters and children’s perspectives were missing, which should be considered in future studies. Furthermore, effects may be inflated because the raters had an investment in the treatment being successful [25]. However, the qualitative feedback seemed to support the findings of the quantitative evaluation.

Although Kidstime workshops were not conceptualized as a therapeutic intervention, the present preliminary findings emphasize that they could have positive therapeutic effects for both children and parents. In the children, there was a significant reduction in impairment and distress. However, parents and practitioners slightly differed: while both observed a moderate improvement in the children`s psychological resilience, the parents observed a strong improvement in their children`s social skills and participation. The practitioners, on the other hand, found that their agency improved significantly. The medium to large effect sizes, reported in the results, indicate the clinical relevance of these findings.

In accordance with these findings, a Spanish research group reported significant pre-post differences after the attendance of Kidstime workshops with regard to improved parental emotional support for their children and reduced self-stigma [26]. Furthermore, we assume that Kidstime workshops work through basic, dignity-based factors that were inadequately reflected in quantitative impact assessment. The qualitative feedback of the families indicated the importance (i.e. ’you are always welcome’, ’meeting on equal terms’, ’be taken seriously in the stresses and strains of family life’, ’you can be who you are’ or ’The feeling that you are understood, no matter what problems you have’).

In another study that evaluated a psychosocial group intervention for COPMI group comparisons failed to show statistically significant intervention effects [27]. A further investigation showed beneficial relationship between social connectedness and adjustment [28]. A systematic review and meta-analysis of twenty trials reported of 50% risk reduction on the incidence of mental disorders in children [29]. In a systematic review [24] five protective factors for COPMI emerged: providing information for children, social support, family functioning and connectedness, child coping, and parenting. As different studies found no association of family functioning with health-related quality of life, problems or psychiatric diagnosis in COPMI [24, 30, 31], we decided not to focus family functioning in the quantitative evaluation of the present study. Nevertheless, our clinical impression was that family connectedness, child coping and parenting were improved through the Kidstime workshops. This was reflected in the qualitative feedback comments (see Supplement 1 Table 3) and in previous studies [11].

In the literature there is a lot of evidence that reliable support made substantial difference in lives of COPMI [28, 29, 32]. Support was related to better health-related quality of life [30], better functioning and less psychopathology, and a decreased likelihood of having a psychiatric disorder [33]. These factors can also be seen in the Kidstime workshops, where all relevant related people are welcome and a consistent team of practitioners is available over a long time.

As regards parents’ mental health, improvements in severity of psychopathology, distress, psychological and social functioning, agency, life satisfaction and resilience were observed. While the quality of life of parents of children with mental illness had been studied [34] and it is known that interdisciplinary support can strengthen parental capabilities and well-being when raising children with developmental disabilities [35], there is almost no literature about the effects of family based interventions on the parents with mental illness.

At various levels of social support, the so-called “social return on investment” needs to be considered, balancing financial expenditures and potential benefits from psychosocial interventions [36]. Parenting interventions for COPMI had positive economic returns through reduced healthcare and education costs and increased productivity [36]. Recent studies indicated a high prevalence of child and adolescent psychiatric disorders among children in residential care [37]. Thus, the comparison of preventive approaches with (non)residential forms of child rearing support can offer important information about intervention effects on children’s well-being and their participation, as well as about cost-effectiveness. Comparing the capability scales of our study with those of other studies (Kips NRW, EVAS), it must be acknowledged that different professionals (social pedagogues, psychologists, doctors) rated these with different professional backgrounds and experiences. This made it difficult to compare case severity. For this reason, Table 3 focused on the change measurement of capabilities to enable some approximate comparison. However, the case numbers of the preventive interventions were lower than those of the youth welfare services and were therefore more prone to error.

Nevertheless, our preliminary results suggest an inverse relationship of the positive clinical effects of preventive approaches in improving capabilities in the children with low number of intervention hours and costs in contrast to child-rearing support. Kidstime workshops showed large effect sizes regarding improvement in psychological integrity and resilience and nearly medium effects regarding participation. The changes in life satisfaction and physical integrity were small. Furthermore, the results indicate that capabilities deteriorate in the most intense and expensive residential care. This could hint at the fact that the educational interventions might be implemented too late and thus had less effects. Nonresidential child rearing support had four times greater costs for less than half of the positive effects. Compared with other preventive interventions, Kidstime workshops showed better results in strengthening mental health, psychological well-being and participation. One explanation could be that Kidstime workshops included the whole family, while parents were not actively involved in the other interventions (KIPS NRW, EVAS). Interventions often take place side by side in an uncoordinated manner in healthcare system. By contrast, Kidstime workshops support the establishment of cross-sectoral network structures for all family members with a mentally ill parent.

One other low-frequency family-oriented intervention for COPMI, CHIMPS (“Children of mentally ill parents”), was based on the early identification of children and young people at risk in psychiatry and child and youth psychiatry [38]. However, only 18.9% of adults with mental illness received guideline-based therapy [39]. There is therefore a danger that the main risk group that does not seek treatment will be overlooked, endangering children with a particularly high risk.

In the Kidstime workshops, some parents also expressed reluctance to participate because of feelings of inadequacy or a fear of being judged as “bad parents”. It is not uncommon for parents to develop strong feelings of guilt and shame, withdraw socially and avoid help for fear of stigma. This is considered a significant risk factor for the emotional well-being of children [40]. Addressing these motivational barriers through sensitive, non-stigmatizing outreach and communication strategies is essential to foster engagement and foster trust among families.

Given the lack of progress in reducing the prevalence, incidence and carer burden of mental illness, a paradigm shift in intervention research and clinical practice is needed [41]. It is important to integrate prevention and treatment interventions including involving non-traditional service providers, improving multidisciplinary collaborations and accessing peer support [42]. Social resilience is based on building a ‘wider self’ [43] by strengthening affiliations, connections and networks.

**Fig. 2:**
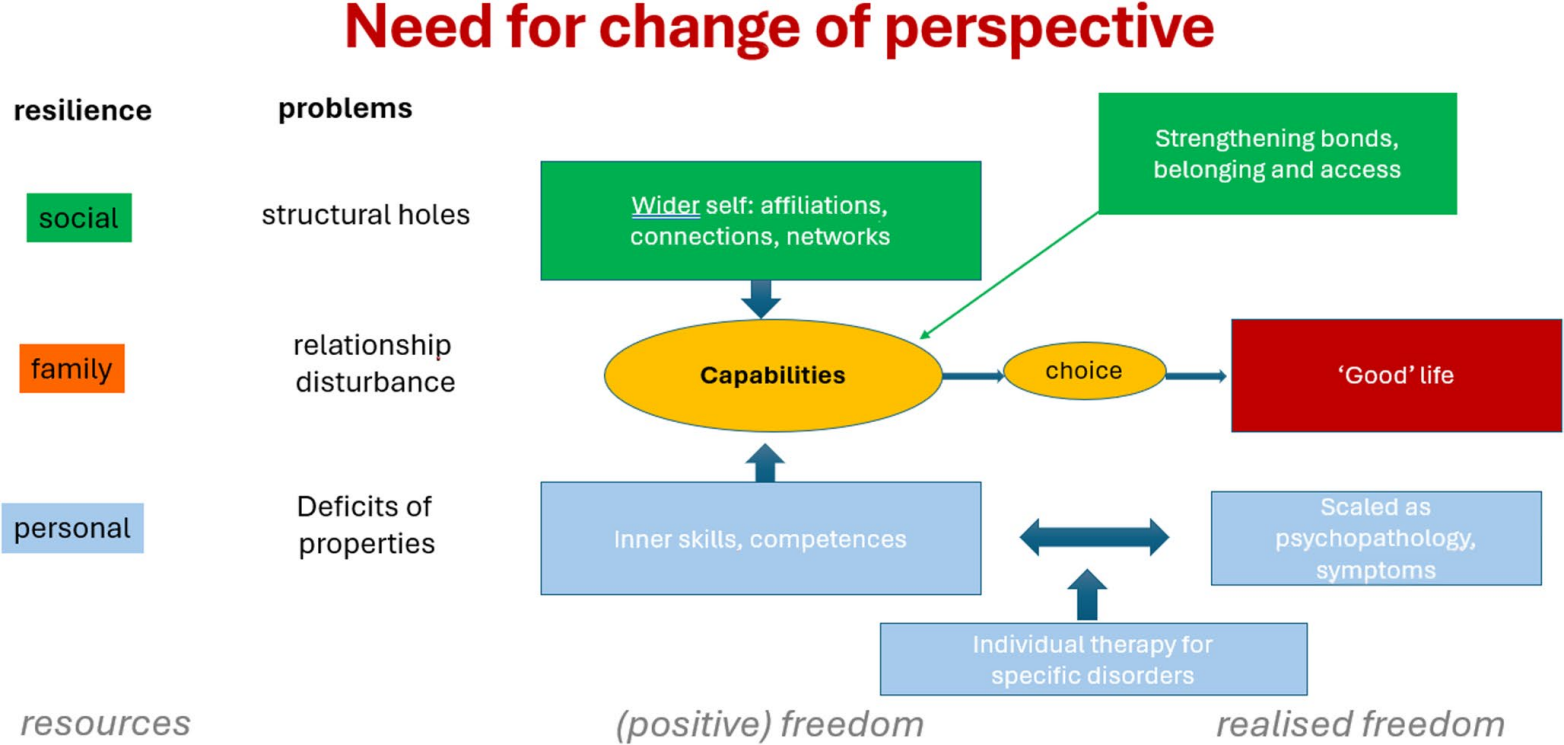
Awakening of capabilities. Framework adapted from [44]

Therefore, a main paradigm shift would be to broaden the perspective (see Fig. 2). In psychotherapy, it is sometimes wrongly assumed that those seeking help have positive freedom to improve their symptoms with the help of disorder-specific interventions. The focus on strengthening personal resilience appears to be missing. Rather, to have the ability to make a choice for a successful life, it is necessary to strengthen capabilities.

Through the multifamily approach Kidstime workshops were more syntonic with, and therefore acceptable to, people’s natural social systems than many other therapeutic interventions. From a clinical perspective, this enabled low-threshold access for families who are often socially disadvantaged [39] and would otherwise not have qualified for a formal therapeutic intervention. From a research perspective, this has made it more difficult to collect a larger dataset and more complete data.

Particularly in times of financial restraints, personnel shortages and increased difficulties in adolescents’ mental health [45] different and more economic approaches to therapy need to be considered. A recent review of 27 intervention trials produced good evidence that nonspecialist providers (peers or community health workers) can successfully deliver treatments with fewer than 10 sessions over 2–3 months in low- and middle-income countries [46]. These and other examples highlight new opportunities in delivering innovative mental health services to effectively and efficiently reduce the burden of mental illness.

Known system-related barriers to access (lack of a common language, poor cooperation and case management, different financing structure bases, etc.) [47] were confirmed by the focus groups. Cooperation with child welfare offices varied widely across regions. Competition for financial resources hindered collaborative efforts. Uncertainties regarding long-term funding created challenges in sustaining Kidstime workshops. This emphasizes the need for greater collaboration and coordination to standardize access for COPMI across local communities. Regarding the access paths to Kidstime workshops, it was striking that there were hardly any referrals from schools and kindergartens. Therefore, future developments should attempt to train staff at schools such as our ‘Mindful Schools’ project, which won the best Erasmus prize for inclusion and diversity in Germany in 2024.

## Conclusion

Our preliminary results indicate that Kidstime workshops can be a low-threshold and cost-effective intervention to promote mental health and participation of COPMI as well as parents’ wellbeing and social resilience.

## Supplementary Information

Below is the link to the electronic supplementary material.

Supplementary Material 1 (DOCX 20.3 KB)

Supplementary Material 2 (DOCX 307 KB)

## Data Availability

No datasets were generated or analysed during the current study.

## Footnotes

1 EVAS is a pedagogic system designed to structure and advance social pedagogic diagnostics and planning in child and youth welfare services. It facilitates methodologically sound evaluations at the individual, institutional, and national levels, offering key insights into structural process and outcome quality. With over 50,000 documented cases, EVAS is the largest quality development tool for educational assistance in Germany and Austria.
