# Supplementary material for: Kidstime workshops: the evaluation of a multi-family intervention for children of parents with mental illness
Source: Eur Child Adolesc Psychiatry. 2025 Aug 27;35(1):263–73. doi: 10.1007/s00787-025-02853-z (PMC12917081; doi:10.1007/s00787-025-02853-z)
Supplement: Supplementary file 1 — Supplementary Material 1 (DOCX 20.3 KB) [file 787_2025_2853_MOESM1_ESM.docx]

# **ECAP, Supplement 1**

Kidstime workshops: the evaluation of a multi-family intervention for children of parents with mental illness

Esther Strittmatter^1*^, Niklas Helsper^2^, Jens Joas^1^, Alan Cooklin^3^, Eva Möhler^1^, Klaus Henner Spierling^4^

^1^Department of Child and Adolescent Psychiatry, Faculty of Medicine, Saarland University, 66421 Homburg, Germany

^2^Integration Assistance and Healthcare Department, Institute for Child and Youth Welfare (IKJ), Essen, Germany

^3^Founder of the Kidstime / OurTime Foundation, London, United Kingdom

^4^Social Pediatric Center, Agaplesion Diakonie Hospital, Rotenburg, Germany

*** Correspondence:** Esther Strittmatter, esther.strittmatter@uks.eu

**Supplement Table 1**: Age of the participants (at T0)

|  | Mean | Standard Deviation | min | max |
| --- | --- | --- | --- | --- |
| Age mothers | 39.44 | 6.20 | 22 | 57 |
| Age fathers | 43.64 | 6.91 | 34 | 58 |
| Age parents | 40.44 | 6.57 | 22 | 58 |
| Age children | 7.36 | 3.77 | 0 | 16 |

**Supplement Table 2:** Diagnosis of the parents and children at T0

|  | Parents | Children |
| --- | --- | --- |
| Disorders due to substance use or addictive behaviours | 7 |  |
| Schizophrenia, schizoaffective disorder | 4 |  |
| Depressive disorders | 53 | 3 |
| Bipolar disorders | 3 |  |
| Anxiety or fear-related disorders | 10 | 4 |
| Disorders of bodily distress or bodily expierience | 3 |  |
| Obsessive-compulsive disorder | 3 |  |
| Eating disorders | 3 | 1 |
| Personality disorder | 14 |  |
| Post-traumatic stress disorder | 8 | 3 |
| Attachment disorder of childhood |  | 3 |
| Conduct disorders |  | 5 |
| Emotional disorders with onset specific to childhood |  | 5 |
| Autism spectrum disorder | 1 | 4 |
| Attention deficit hyperactivity disorder | 4 | 13 |
| Developmental learning disorder |  | 3 |
| Disorders of intellectual developement |  | 1 |

**Supplement Table 3:** Examples from the qualitative feedback evaluation regarding family connectedness, child coping and parenting

| ’great cohesion’, ’have a great time together’, ’great ideas for home’, ’conscious time for children and their feelings’, ’exchange ideas and feel good with like-minded people’, ’good advice for myself’, ’make new friends’, ‘new ideas for interacting with children’, ’take a break from everyday life and spend time with the family’, ’a carefree time together’, ’the children feel understood’ or ’children find new ways to express themselves in the theatre’ and ’now, I understand better, what my child needs’ |
| --- |
