# Supplementary material for: Kidstime workshops: the evaluation of a multi-family intervention for children of parents with mental illness
Source: Eur Child Adolesc Psychiatry. 2025 Aug 27;35(1):263–73. doi: 10.1007/s00787-025-02853-z (PMC12917081; doi:10.1007/s00787-025-02853-z)

# **ECAP,Supplement 2**

Kidstime workshops: the evaluation of a multi-family intervention for children of parents with mental illness

Esther Strittmatter^1*^, Niklas Helsper^2^, Jens Joas^1^, Alan Cooklin^3^, Eva Möhler^1^, Klaus Henner Spierling^4^

^1^Department of Child and Adolescent Psychiatry, Faculty of Medicine, Saarland University, 66421 Homburg, Germany

^2^Integration Assistance and Healthcare Department, Institute for Child and Youth Welfare (IKJ), Essen, Germany

^3^Founder of the Kidstime / OurTime Foundation, London, United Kingdom

^4^Social Pediatric Center, Agaplesion Diakonie Hospital, Rotenburg, Germany

*** Correspondence:** Esther Strittmatter, esther.strittmatter@uks.eu

**Distribution Analysis of the MANOVA data set/ final sample size:**

1. **Parent data set – Parents CAP 1, 2, 3, 10 in self-assessment**

n = 50

For each of the 4 Caps, a population pyramid comparing the frequencies of the given answers and a summary comparison in the form of a line chart with all relevant CAP scales.

The response values were defined as follows:

0 = not true at all

1 = less true

2 = not really true

3 = somewhat true

4 = mostly true

5 = completely true

**Fig. 1:** Population pyramid of Cap1 response frequencies in parents' self-assessment (n=50):


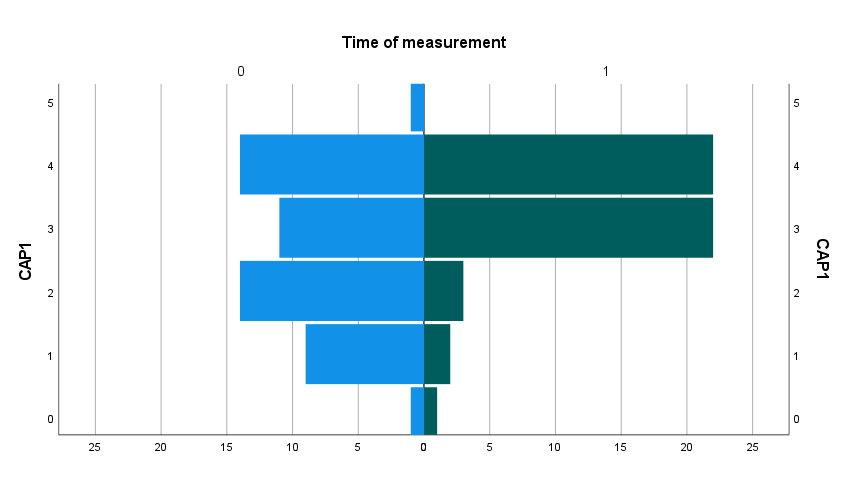


**Fig. 2:** Population pyramid of response frequencies Cap2 parents' self-assessment (n=50)


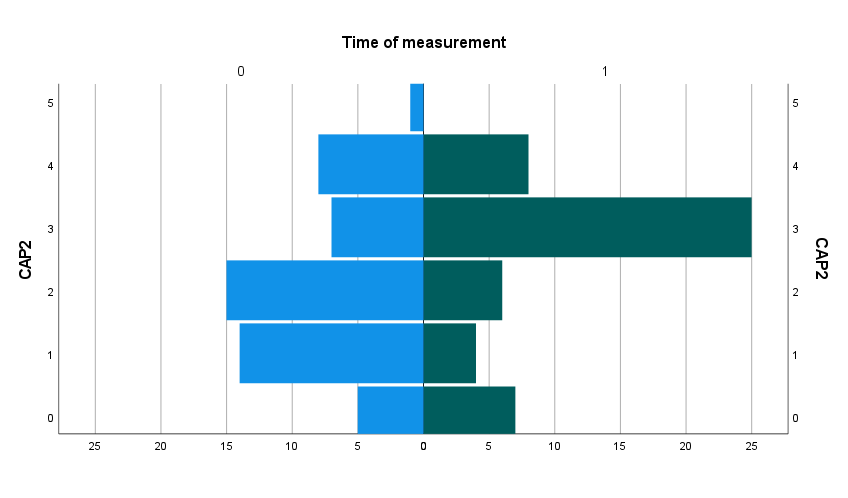


**Fig.3:** Population pyramid of response frequencies Cap 3 Parents' self-assessment (n=50)


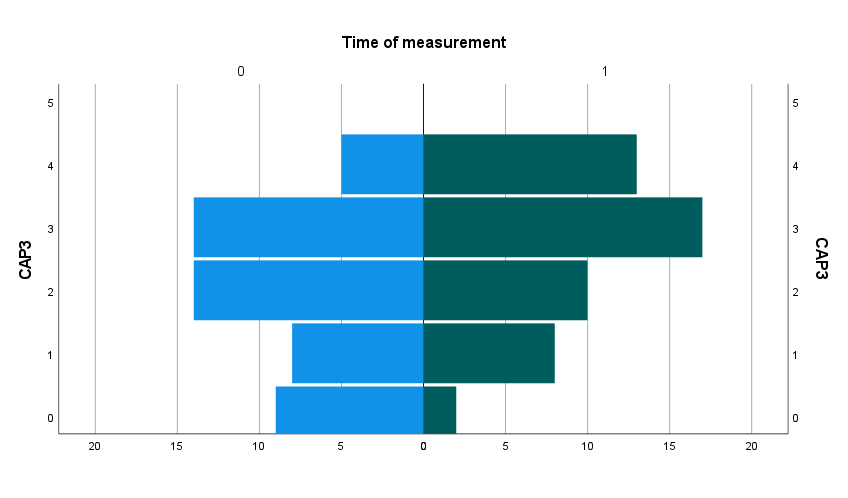


**Fig. 4:** Population pyramid of response frequencies Cap 10 Parents' self-assessment (n=50)


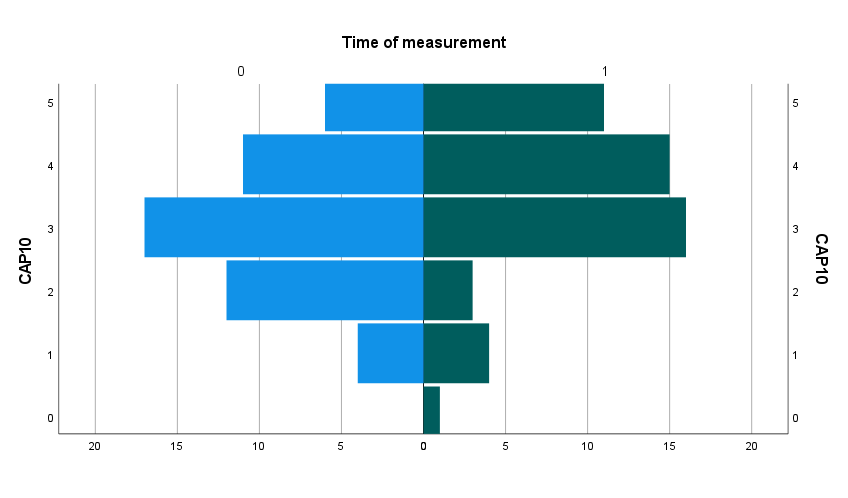


**Tab. 1:** Mean values (M), standard deviation (SD), change (+/-) from T0 to T1 and minimum (Min)/ maximum (Max) response values of the parental self assessment

| Parent data | n | *M* | *SD* | +/- | Min. | Max. |
| --- | --- | --- | --- | --- | --- | --- |
| CAP1 T0 | 50 | 2.62 | 1.19 | +.62 | 0 | 5 |
| CAP1 T1 | 50 | 3.24 | .89 |  | 0 | 4 |
| CAP2 T0 | 50 | 2.04 | 1.29 | +.42 | 0 | 5 |
| CAP2 T1 | 50 | 2.46 | 1.27 |  | 0 | 4 |
| CAP3 T0 | 50 | 1.96 | 1.26 | +.66 | 0 | 4 |
| CAP3 T1 | 50 | 2.62 | 1.16 |  | 0 | 4 |
| CAP10 T0 | 50 | 3.06 | 1.13 | +.40 | 1 | 5 |
| CAP10 T1 | 50 | 3.46 | 1.25 |  | 0 | 5 |

**Fig. 5:** Line graph showing the mean values of the respective cap scale parents' self-assessment (n=50)


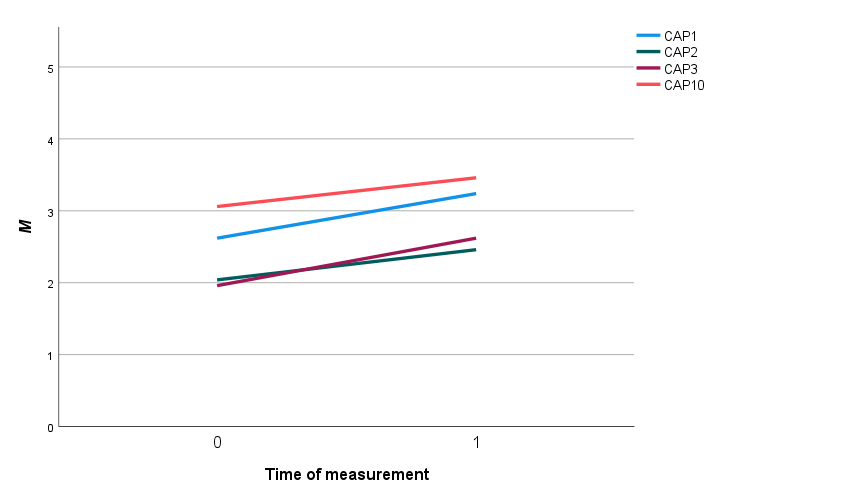


1. **Parent data set – Cap 1, 2, 3, 10 professionals referring to parents**

n = 31

For each of the four Caps, a population pyramid comparing the frequencies of the answers given and a summary comparison in the form of a line chart with all relevant Cap scales.

The response values were defined as follows:

0 = not true at all; 1 = somewhat true; 2 = not really true; 3 = somewhat true;
4 = mostly true; 5 = completely true

**Fig. 6:** Population pyramid of response frequencies Cap1 professionals in relation to parents (n=31)


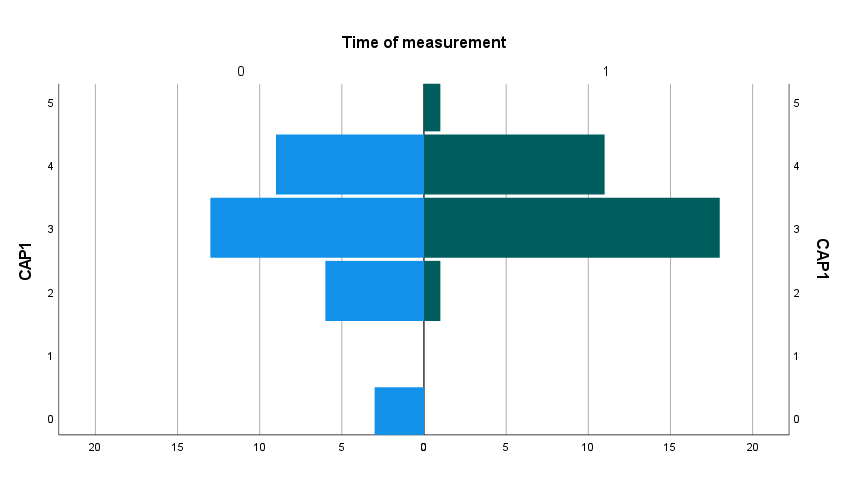


**Fig. 7:** Population pyramid of response frequencies Cap2 professionals in relation to parents (n=31)


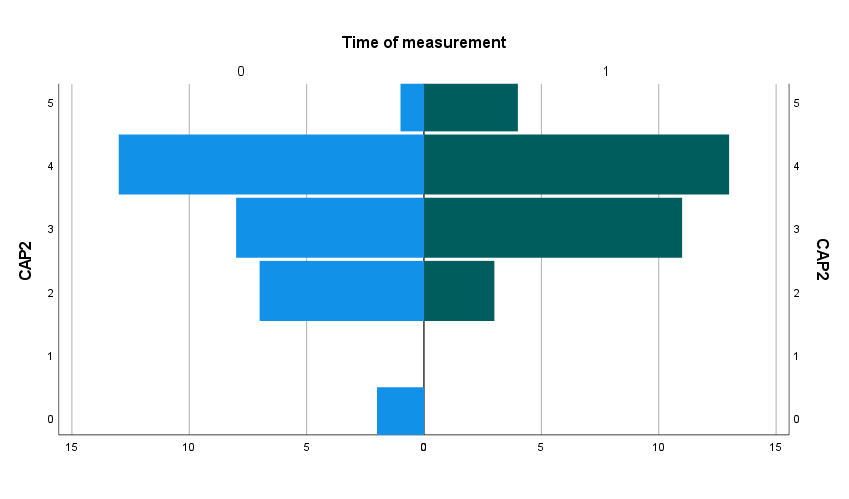


**Fig. 8:** Population pyramid of response frequencies Cap3 professionals in relation to parents (n=31)


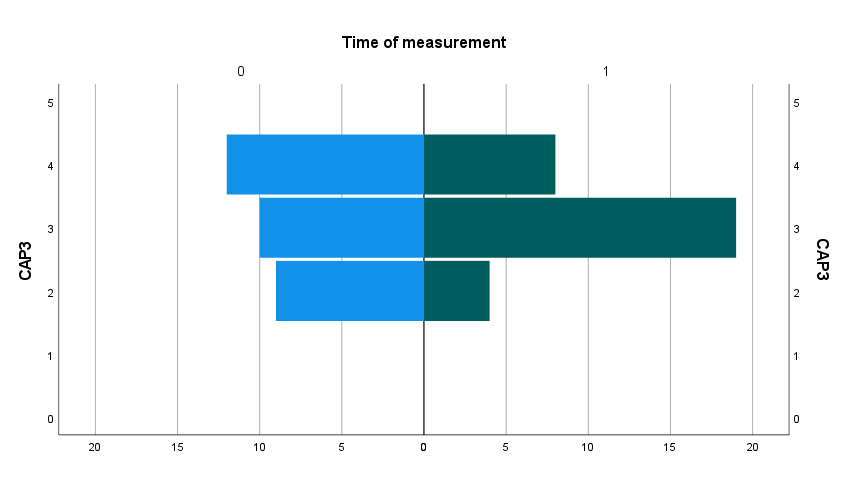


**Fig. 9:** Population pyramid of response frequencies Cap10 professionals in relation to parents (n=31)


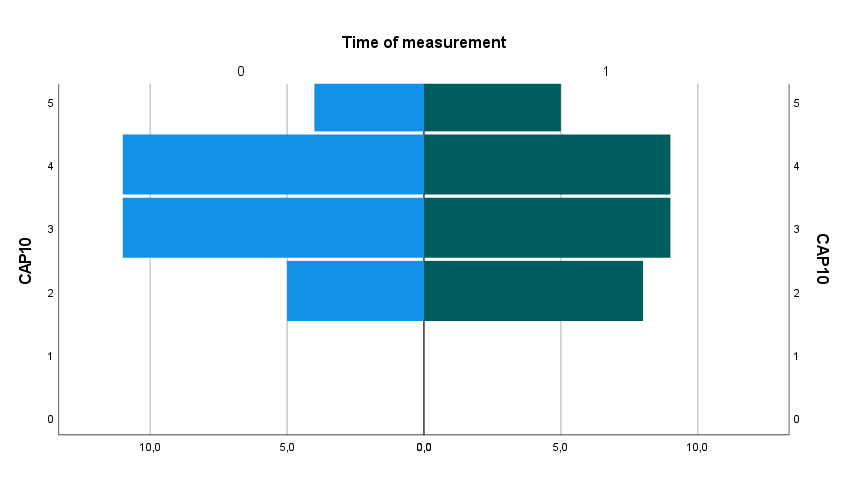


**Tab. 2:** Mean values (M), standard deviation (SD), change (+/-) from T0 to T1 and minimum (Min)/ maximum (Max) response values (professionals in relation to parents)

| Professionals data | n | *M* | *SD* | +/- | Min. | Max. |
| --- | --- | --- | --- | --- | --- | --- |
| CAP1 T0 | 31 | 2.81 | 1.17 | +.58 | 0 | 4 |
| CAP1 T1 | 31 | 3.39 | .62 |  | 2 | 5 |
| CAP2 T0 | 31 | 3.06 | 1.18 | +.52 | 0 | 5 |
| CAP2 T1 | 31 | 3.58 | .850 |  | 2 | 5 |
| CAP3 T0 | 31 | 3.10 | .83 | +.03 | 2 | 4 |
| CAP3 T1 | 31 | 3.13 | .62 |  | 2 | 4 |
| CAP10 T0 | 31 | 3.45 | .93 | -.10 | 2 | 5 |
| CAP10 T1 | 31 | 3.35 | 1.05 |  | 2 | 5 |

**Fig. 10:** Line chart showing the mean values of the respective cap scale for professionals in relation to the parents (n=31)


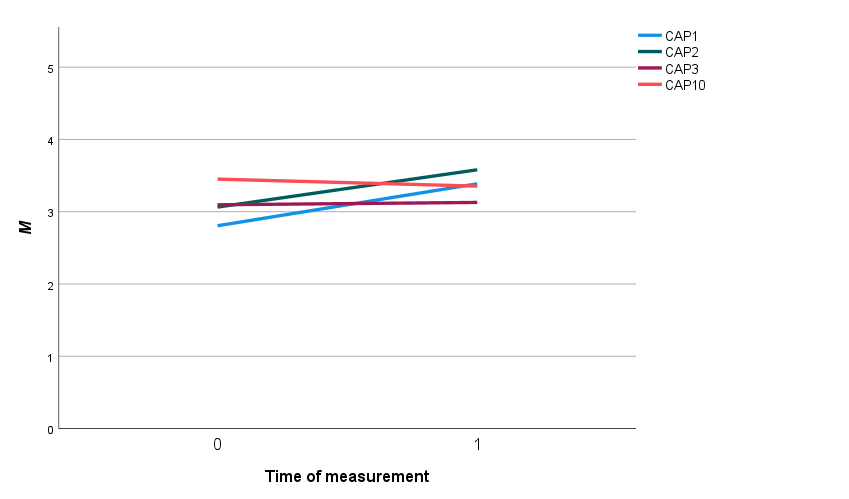


1. **Parents Cap 1,2,3,10 in relation to their child**

n = 55

For each of the 4 Caps, a population pyramid comparing the frequencies of the given answers and a summary comparison in the form of a line chart with all relevant Cap scales. The response values were defined as follows:

0 = not true at all

1 = less true

2 = not really true

3 = somewhat true

4 = mostly true

5 = completely true

**Fig. 11:** Population pyramid of response frequencies Cap1 parents in relation to their child (n=55)


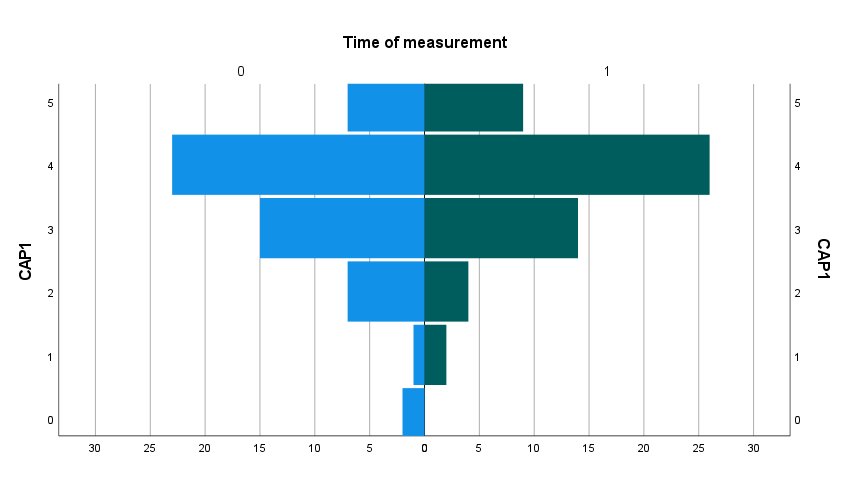


**Fig. 12:** Population pyramid of response frequencies Cap2 parents in relation to their child (n=55)


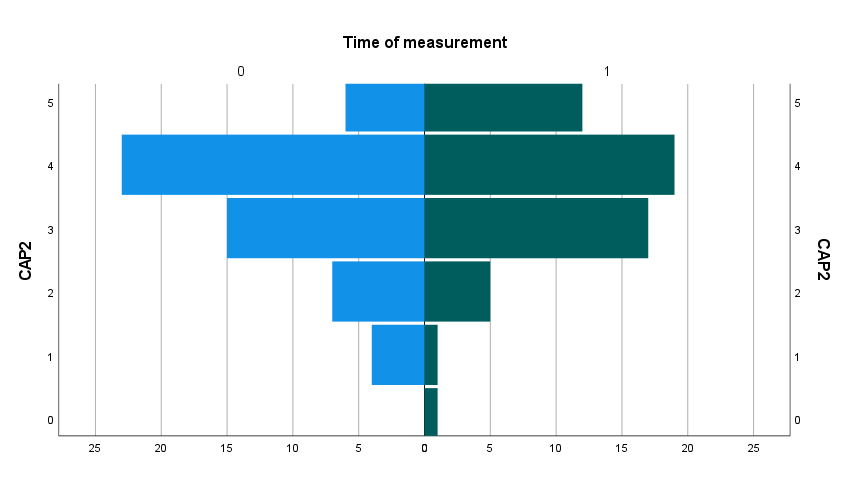


**Fig. 13:** Population pyramid of response frequencies Cap3 parents in relation to their child (n=55)


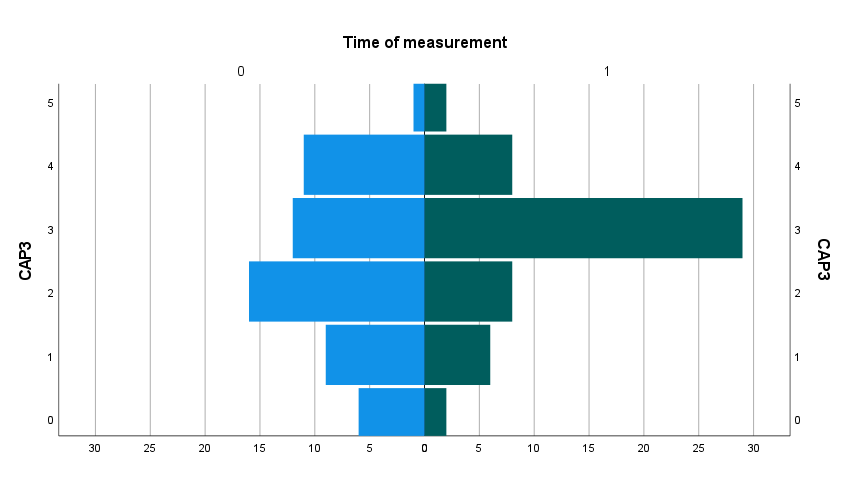


**Fig. 14:** Population pyramid of response frequencies Cap10 parents in relation to their child (n=55)


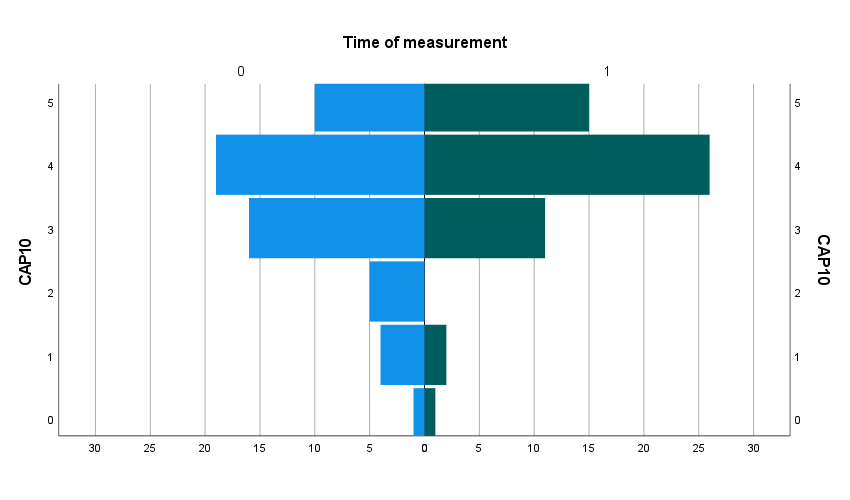


**Tab. 3:** Mean values (M), standard deviation (SD), change (+/-) from T0 to T1 and minimum (Min)/ maximum (Max) response values (parents in relation to their child)

| Parent data | n | *M* | *SD* | +/- | Min. | Max. |
| --- | --- | --- | --- | --- | --- | --- |
| CAP1 T0 | 55 | 3.40 | 1.15 | +.25 | 0 | 5 |
| CAP1 T1 | 55 | 3.65 | .97 |  | 1 | 5 |
| CAP2 T0 | 55 | 3.36 | 1.08 | +.24 | 1 | 5 |
| CAP2 T1 | 55 | 3.60 | 1.10 |  | 0 | 5 |
| CAP3 T0 | 55 | 2.29 | 1.31 | +.46 | 0 | 5 |
| CAP3 T1 | 55 | 2.75 | 1.08 |  | 0 | 5 |
| CAP10 T0 | 55 | 3.42 | 1.21 | +.47 | 0 | 5 |
| CAP10 T1 | 55 | 3.89 | 1.05 |  | 0 | 5 |

**Fig. 15:** Line graph showing the mean values of the respective Cap scale for parents in relation to their child (n=55)


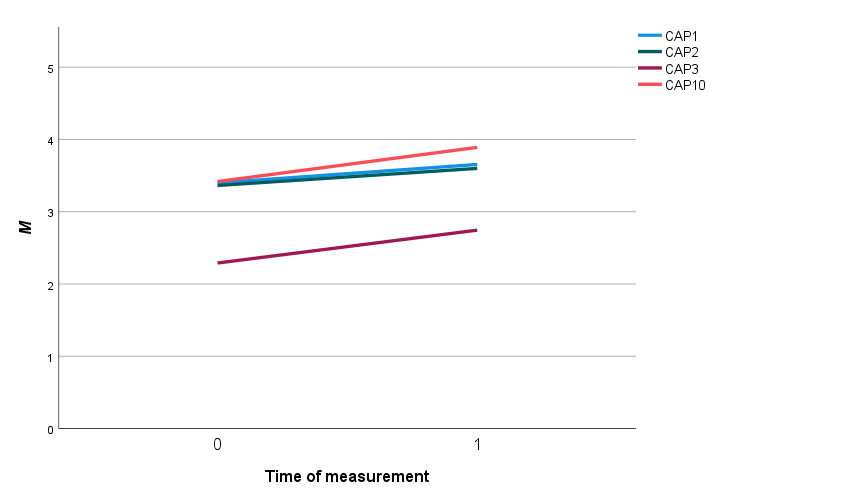


1. **Professionals Cap 1,2,3,10 per child**

n = 32

For each of the 4 Caps, a population pyramid comparing the frequencies of the given answers and a summary comparison in the form of a line chart with all relevant Cap scales. The answer values were defined as follows:

0 = not true at all

1 = less true

2 = rather not true

3 = rather true

4 = mostly true

5 = completely true

**Fig. 16:** Population pyramid of response frequencies Cap1 professionals in relation to children (n=32)


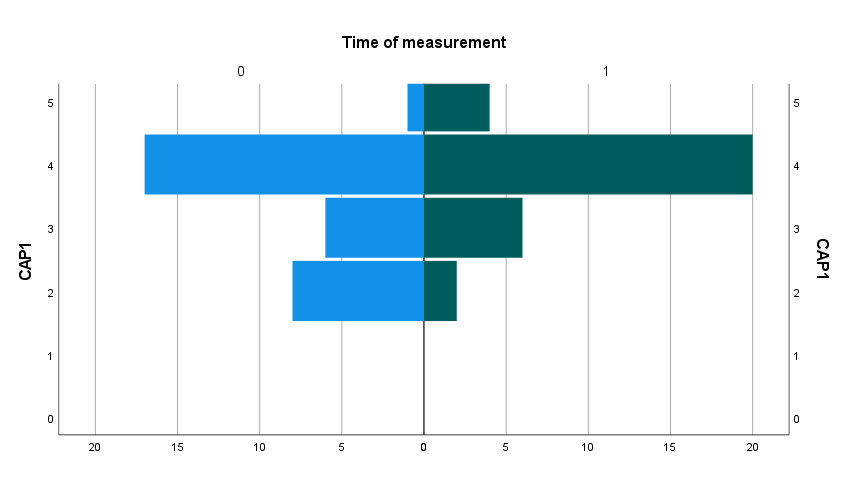


**Fig. 17:** Population pyramid of response frequencies Cap2 professionals in relation to children (n=32)


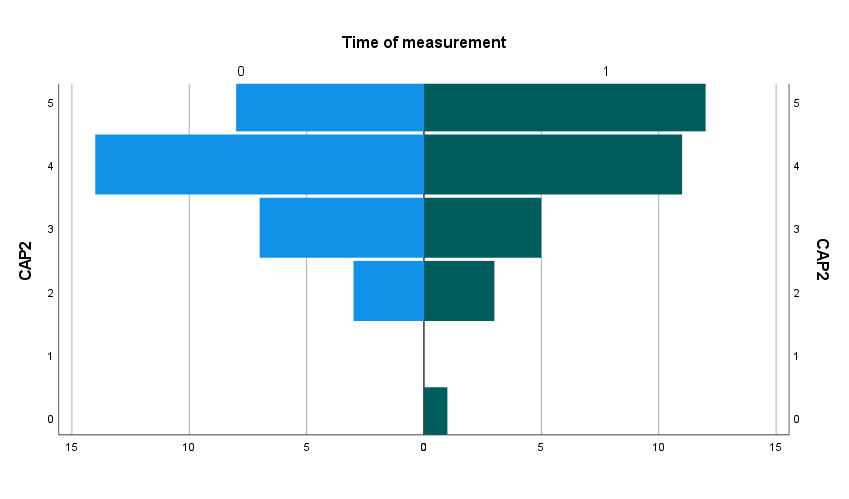


**Fig. 18:** Population pyramid of response frequencies Cap3 professionals in relation to children (n=32)


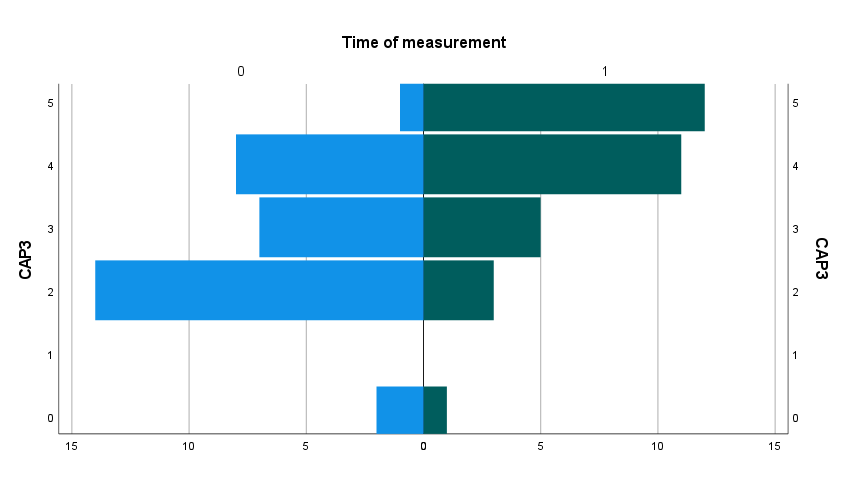


**Fig. 19:** Population pyramid of response frequencies Cap10 professionals in relation to children (n=32)


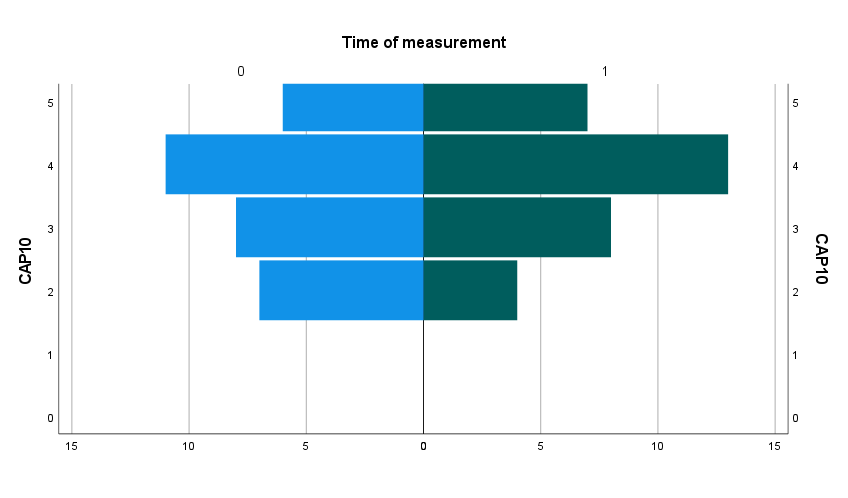


**Tab. 4:** Mean values (M), standard deviation (SD), change (+/-) from T0 to T1 and minimum (Min)/ maximum (Max) response values (professionals in relation to their child)

| Professionals data | n | *M* | *SD* | +/- | Min. | Max. |
| --- | --- | --- | --- | --- | --- | --- |
| CAP1 T0 | 32 | 3.34 | .90 | +.47 | 2 | 5 |
| CAP1 T1 | 32 | 3.81 | .74 |  | 2 | 5 |
| CAP2 T0 | 32 | 3.84 | .92 | +.07 | 2 | 5 |
| CAP2 T1 | 32 | 3.91 | 1.20 |  | 0 | 5 |
| CAP3 T0 | 32 | 2.69 | 1.15 | +1.22 | 0 | 5 |
| CAP3 T1 | 32 | 3.91 | 1.20 |  | 0 | 5 |
| CAP10 T0 | 32 | 3.50 | 1.05 | +.22 | 2 | 5 |
| CAP10 T1 | 32 | 3.72 | .96 |  | 2 | 5 |

**Fig. 20:** Line chart showing the mean values of the respective Cap scale for professionals in relation to children (n=32)


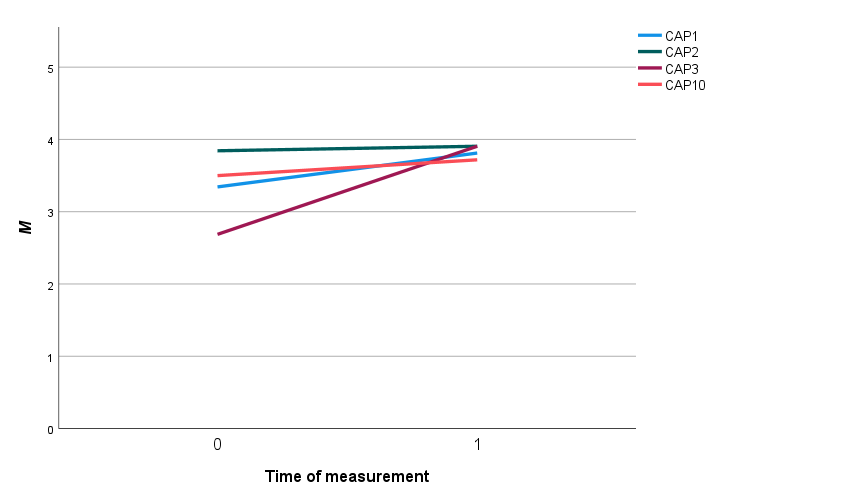


**Distribution Analysis of the data set of Table 3:**

|  | | | | | |
| --- | --- | --- | --- | --- | --- |
|  | | *Cap 1 Children* | *Cap 2 Children* | *Cap 3 Children* | *Cap 10 Children* |
| *N* | *Valid* | **54** | **53** | **54** | **50** |
| *Mean* | | **5,3086** | **3,8679** | **14,2284** | **8,1000** |
| *Median* | | **5,8333** | **,0000** | **20,0000** | **8,3333** |
| *Std. Deviation* | | **17,98285** | **15,45870** | **21,67202** | **22,60027** |
| *Minimum* | | **-30,00** | **-30,00** | **-40,00** | **-46,67** |
| *Maximum* | | **50,00** | **40,00** | **80,00** | **60,00** |
| *Percentiles* | *25* | **-7,5000** | **-6,6667** | **,0000** | **-6,6667** |
|  | *50* | **5,8333** | **,0000** | **20,0000** | **8,3333** |
|  | *75* | **20,0000** | **17,5000** | **26,6667** | **20,0000** |
| **Change measurement Cap 1 Children** | | | |  |  |
|  | | *Frequency* | *Valid Percent* | *Cumulative Percent* |  |
| *Valid* | *-30,00* | **2** | **3,7** | **3,7** |  |
|  | *-20,00* | **6** | **11,1** | **14,8** |  |
|  | *-13,33* | **3** | **5,6** | **20,4** |  |
|  | *-10,00* | **2** | **3,7** | **24,1** |  |
|  | *-6,67* | **1** | **1,9** | **25,9** |  |
|  | *,00* | **11** | **20,4** | **46,3** |  |
|  | *3,33* | **1** | **1,9** | **48,1** |  |
|  | *5,00* | **1** | **1,9** | **50,0** |  |
|  | *6,67* | **3** | **5,6** | **55,6** |  |
|  | *10,00* | **5** | **9,3** | **64,8** |  |
|  | *13,33* | **4** | **7,4** | **72,2** |  |
|  | *16,67* | **1** | **1,9** | **74,1** |  |
|  | *20,00* | **8** | **14,8** | **88,9** |  |
|  | *25,00* | **1** | **1,9** | **90,7** |  |
|  | *30,00* | **1** | **1,9** | **92,6** |  |
|  | *40,00* | **3** | **5,6** | **98,1** |  |
|  | *50,00* | **1** | **1,9** | **100,0** |  |
|  | *Total* | **54** | **100,0** |  |  |


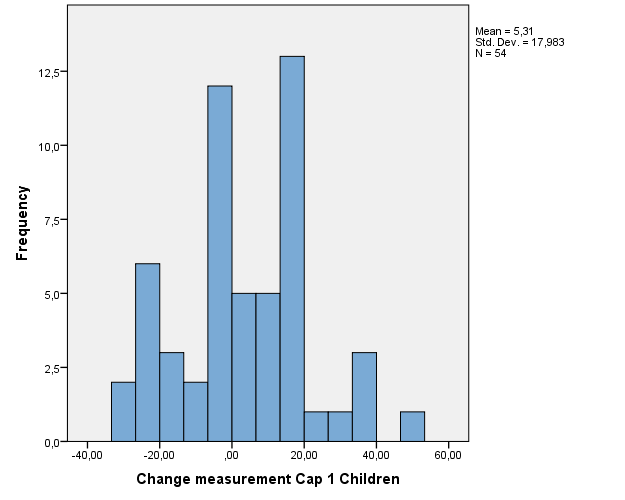


| **Change measurement Cap 2 Children** | | | |  |
| --- | --- | --- | --- | --- |
|  | | *Frequency* | *Valid Percent* | *Cumulative Percent* |
| *Valid* | *-30,00* | **1** | **1,9** | **1,9** |
|  | *-26,67* | **1** | **1,9** | **3,8** |
|  | *-20,00* | **5** | **9,4** | **13,2** |
|  | *-10,00* | **3** | **5,7** | **18,9** |
|  | *-6,67* | **4** | **7,5** | **26,4** |
|  | *-5,00* | **1** | **1,9** | **28,3** |
|  | *,00* | **13** | **24,5** | **52,8** |
|  | *3,33* | **1** | **1,9** | **54,7** |
|  | *5,00* | **1** | **1,9** | **56,6** |
|  | *6,67* | **2** | **3,8** | **60,4** |
|  | *10,00* | **5** | **9,4** | **69,8** |
|  | *13,33* | **2** | **3,8** | **73,6** |
|  | *15,00* | **1** | **1,9** | **75,5** |
|  | *20,00* | **9** | **17,0** | **92,5** |
|  | *30,00* | **3** | **5,7** | **98,1** |
|  | *40,00* | **1** | **1,9** | **100,0** |
|  | *Total* | **53** | **100,0** |  |


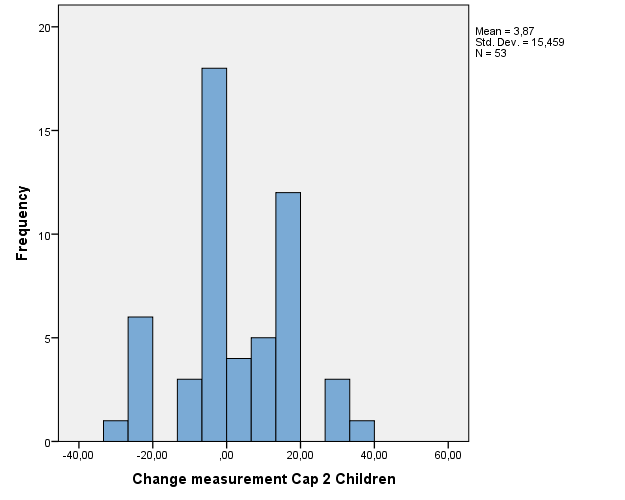


| **Change measurement Cap 3 Children** | | | |  |
| --- | --- | --- | --- | --- |
|  | | *Frequency* | *Valid Percent* | *Cumulative Percent* |
| *Valid* | *-40,00* | **2** | **3,7** | **3,7** |
|  | *-20,00* | **2** | **3,7** | **7,4** |
|  | *-13,33* | **1** | **1,9** | **9,3** |
|  | *-10,00* | **2** | **3,7** | **13,0** |
|  | *-6,67* | **1** | **1,9** | **14,8** |
|  | *,00* | **9** | **16,7** | **31,5** |
|  | *6,67* | **3** | **5,6** | **37,0** |
|  | *10,00* | **4** | **7,4** | **44,4** |
|  | *20,00* | **16** | **29,6** | **74,1** |
|  | *26,67* | **3** | **5,6** | **79,6** |
|  | *30,00* | **3** | **5,6** | **85,2** |
|  | *33,33* | **1** | **1,9** | **87,0** |
|  | *35,00* | **1** | **1,9** | **88,9** |
|  | *40,00* | **3** | **5,6** | **94,4** |
|  | *50,00* | **1** | **1,9** | **96,3** |
|  | *60,00* | **1** | **1,9** | **98,1** |
|  | *80,00* | **1** | **1,9** | **100,0** |
|  | *Total* | **54** | **100,0** |  |


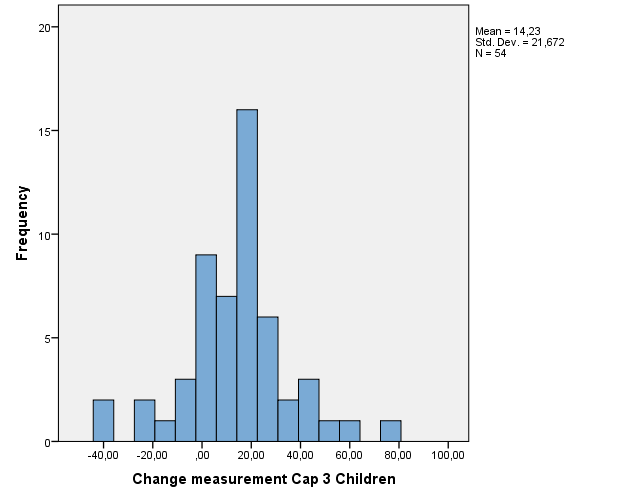


| **Change measurement Cap 10 Children** | | | |  |
| --- | --- | --- | --- | --- |
|  | | *Frequency* | *Valid Percent* | *Cumulative Percent* |
| *Valid* | *-46,67* | **1** | **2,0** | **2,0** |
|  | *-40,00* | **1** | **2,0** | **4,0** |
|  | *-26,67* | **1** | **2,0** | **6,0** |
|  | *-20,00* | **3** | **6,0** | **12,0** |
|  | *-16,67* | **1** | **2,0** | **14,0** |
|  | *-13,33* | **1** | **2,0** | **16,0** |
|  | *-10,00* | **3** | **6,0** | **22,0** |
|  | *-6,67* | **2** | **4,0** | **26,0** |
|  | *,00* | **10** | **20,0** | **46,0** |
|  | *3,33* | **1** | **2,0** | **48,0** |
|  | *6,67* | **1** | **2,0** | **50,0** |
|  | *10,00* | **3** | **6,0** | **56,0** |
|  | *13,33* | **3** | **6,0** | **62,0** |
|  | *20,00* | **10** | **20,0** | **82,0** |
|  | *25,00* | **2** | **4,0** | **86,0** |
|  | *26,67* | **1** | **2,0** | **88,0** |
|  | *40,00* | **2** | **4,0** | **92,0** |
|  | *45,00* | **1** | **2,0** | **94,0** |
|  | *50,00* | **1** | **2,0** | **96,0** |
|  | *60,00* | **2** | **4,0** | **100,0** |
|  | *Total* | **50** | **100,0** |  |


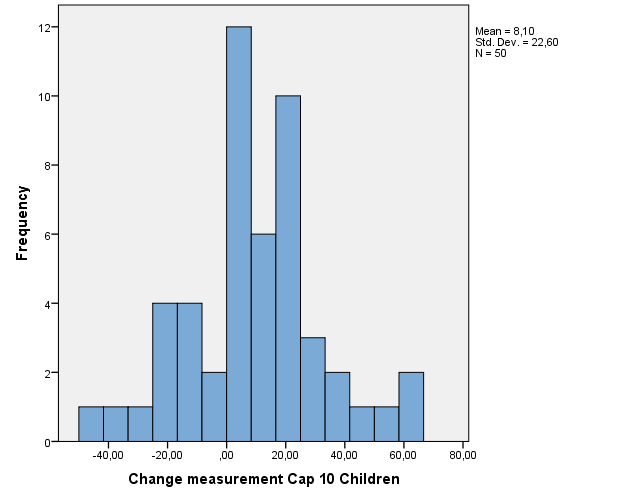

Supplement: Supplementary file 2 — Supplementary Material 2 (DOCX 307 KB) [file 787_2025_2853_MOESM2_ESM.docx]
